# Analysis techniques in the presence of non-proportional hazards (PH); application to an ovarian cancer RCT with long-term follow-up

**DOI:** 10.1186/1745-6215-16-S2-P141

**Published:** 2015-11-16

**Authors:** James Morden, Judith Bliss, Rosalind Eeles

**Affiliations:** 1 Clinical Trials and Statistics Unit, The Institue of Cancer Research, London, UK; 2 Oncogenetics Team, The Institute of Cancer Research, London, UK

## Introduction

AHT was an international, non-blinded phase III RCT designed to assess whether administering adjuvant hormone therapy (AHT) after ovarian cancer affects overall survival (OS). Between 1990-1995, 150 patients were randomised to receive AHT (n=75) or no AHT (Control, n=75). Death data for UK patients (83% of total) provided by the Health and Social Care Information Centre (HSCIC) has allowed long-term follow-up and therefore detailed investigation into the pattern of events over time. At time of data snapshot, median follow-up was 19yrs.

## Methods

Initially, OS analyses were planned using the Cox-PH model; however there was clear violation of the PH assumption when assessed using graphical techniques and Schoenfeld residuals. Absolute difference between survival estimates in each group was plotted against time and compared to inclusion of a time-dependent covariate in a Cox-PH model. Restricted Mean Survival (RSM) was calculated, using a pre-defined t* of 20yrs. Sensitivity of results to choice of t* was explored.

## Results

Comparison of PH test results between sequential analyses suggested violation of the assumption was sensitive to late emerging data (Mar-10:p=0.19; Sep-12:p=0.048). RSM at 20yrs was AHT: 8.5yrs vs. Control: 5.7yrs, absolute difference 2.8yrs (95%CI 0.3-5.2). Absolute difference varied to 1.0yrs (95%CI -0.3-2.3) with t*=10 and 1.8yrs (95%CI -0.1-3.8) with t*=15.

## Conclusions

Graphical display of difference in survival estimates over time allows insight into patterns of events in studies with long-term follow-up. RSM is an effective method for analysing survival data in the presence of non-PH, however choice of t* may affect robustness of results.

